# Global Transcriptomic Changes Elicited by *sodB* Deletion and Menadione Exposure in *Aspergillus nidulans*

**DOI:** 10.3390/jof9111060

**Published:** 2023-10-30

**Authors:** Klaudia Pákozdi, Tamás Emri, Károly Antal, István Pócsi

**Affiliations:** 1 Department of Molecular Biotechnology and Microbiology, Institute of Biotechnology, Faculty of Science and Technology, University of Debrecen, H-4032 Debrecen, Hungary; pakozdi.klaudia@science.unideb.hu; 2 HUN-REN–UD Fungal Stress Biology Research Group, H-4032 Debrecen, Hungary; 3 Doctoral School of Nutrition and Food Sciences, University of Debrecen, H-4032 Debrecen, Hungary; 4 Department of Zoology, Eszterházy Károly Catholic University, H-3300 Eger, Hungary; antalk2@gmail.com

**Keywords:** *Aspergillus nidulans*, menadione stress, mitochondrial superoxide dismutase, RNA sequencing, SodB

## Abstract

Manganese superoxide dismutases (MnSODs) play a pivotal role in the preservation of mitochondrial integrity and function in fungi under various endogenous and exogenous stresses. Deletion of *Aspergillus nidulans* mnSOD/SodB increased oxidative stress sensitivity and apoptotic cell death rates as well as affected antioxidant enzyme and sterigmatocystin productions, respiration, conidiation and the stress tolerance of conidiospores. The physiological consequences of the lack of *sodB* were more pronounced during carbon starvation than in the presence of glucose. Lack of SodB also affected the changes in the transcriptome, recorded by high-throughput RNA sequencing, in menadione sodium bisulfite (MSB)-exposed, submerged cultures supplemented with glucose. Surprisingly, the difference between the global transcriptional changes of the *ΔsodB* mutant and the control strain were relatively small, indicating that the SodB-dependent maintenance of mitochondrial integrity was not essential under these experimental conditions. Owing to the outstanding physiological flexibility of the Aspergilli, certain antioxidant enzymes and endogenous antioxidants together with the reduction in mitochondrial functions compensated well for the lack of SodB. The lack of *sodB* reduced the growth of surface cultures more than of the submerged culture, which should be considered in future development of fungal disinfection methods.

## 1. Introduction

Superoxide dismutases (SODs) are ubiquitous, ancient metalloenzymes present in both prokaryotes and eukaryotes that dismutate superoxide anion radicals (O_2_^●−^) into molecular oxygen (O_2_) and hydrogen peroxide (H_2_O_2_) and, hence, they are basic elements of oxidative stress defense [1,2]). Manganese-SODs (MnSODs) are primarily localized in the mitochondria of yeasts and filamentous fungi and are key players in the maintenance of mitochondrial integrity and functions [2,3,4,5].

The physiological consequences of the ablation of MnSOD-encoding genes have been studied in detail in some model organisms, including mapping global gene expression changes compensating for the loss of this important mitochondrial enzyme.

For example, in exponential-growth-phase *Saccharomyces cerevisiae* cultures, deletion of POS5 (coding for the mitochondrial NADH kinase), *SOD1* (encodes the cytoplasmic CuZnSOD) or *SOD2* (the MnSOD enzyme gene) results in surprisingly similar global transcriptomic changes reflecting the downregulation of a number of oxidative stress response genes in addition to amino acid metabolism, carbohydrate metabolism, RNA and DNA metabolism, transport, protein folding, etc. processes [6]. Furthermore, *POS5* mutants show upregulation of many genes associated with anaerobic/hypoxic growth. In conclusion, mutant cells seem to respond to altered (reduced) mitochondrial activity and not directly to elevations in intracellular ROS concentrations [6].

Most recently, it was also demonstrated that *S. cerevisiae* cells shut down respiration under paraquat exposures to reduce harmful intramitochondrial O_2_^●−^ production via genetically controlled deletion of mitochondrial oxidative phosphorylation genes [7]. This swift adaptation process relies on Sod2 activity as well as on two-way mitochondrial-nuclear communication through Rtg2 (encoding a sensor of mitochondrial dysfunction) and Rtg3 (transcription factor for retrograde pathway) [7].

Interestingly, deletion of the *Pasod3* gene encoding MnSOD does not affect the life span of the biological aging model organism *Podospora anserina* because the disadvantageous physiological changes (high intracellular O_2_^●−^ levels, impaired Complex I-dependent respiration) is mitigated by upregulated autophagy, especially mitophagy [8]. Importantly, lack of PaSod3 triggers a global transcriptional response via upregulating genes coding for components of mitochondrial processes involved in mitochondrial biogenesis, mitochondrial metabolism or mitochondrial respiration [8].

In the filamentous fungus model organism *Aspergillus nidulans*, deletion of the *mnSOD/sodB* gene negatively affects menadione and PAF (*Penicillium chrysogenum* antifungal protein, eliciting apoptotic cell death in sensitive fungi) tolerances, increases intracellular glutathione reductase and catalase activities (in growing cultures) and sterigmatocystin production (in starving cultures), lowers total, KCN-sensitive cytochrome c-dependent and AOX-dependent alternative respirations while increasing KCN + SHAM (salicyl hydroxamate, which blocks alternative oxidase)-resistant, residual respiration (in carbon-starving cultures), decreases KCN + SHAM-resistant respiration in glucose-supplemented cultures and reduces conidia yields as well as decreases heat (50 °C) tolerance and viability (stored at 4 °C) of conidia [5]. The *ΔmnSOD/ΔsodB* strain grows more slowly with 4 h of incubation time when transferred to a fresh, glucose-supplemented culture medium, but this delayed growth phenotype disappears when the incubation time is extended up to 10 h [5].

It is important to note that *sodB* orthologous genes have been functionally characterized in other Aspergilli as well. For example, *Afsod2* coding for the mitochondrial MnSOD of the opportunistic human pathogenic fungus *Aspergillus fumigatus* is highly expressed in conidia and in growing mycelium at later time points and is inducible by menadione and heat treatments [9]. As expected, the ablation of *Afsod2* results in superoxide and heat-sensitive phenotypes [9]. The *Aspergillus flavus Δsod* (mnSOD deleted strain) mutant grows more slowly than the wild-type strain under all culture conditions tested and also produces a lower amount of aflatoxin B1 in vivo when grown on maize ears in the field [10]. Interestingly, cumene hydroperoxide treatment d0es not influence the growth of the *Δsod* mutant but decreases its aflatoxin B1 production in static Czapek-Dox medium [10].

Considering the outstanding industrial, biomedical and agricultural importance of the Aspergilli, we aimed at the in-depth analysis of the global transcriptome changes provoked by *sodB* gene deletion and menadione (as a superoxide-generating compound) exposure in submerged liquid cultures of *A. nidulans*. As a result, we shed light on the role of SodB in the menadione stress response and also on the effective compensatory mechanisms, which were set into operation in the absence of the *sodB* gene. We hope that these new observations will be exploitable in future antifungal drug research and in future industrial strain developments as well.

## 2. Materials and Methods

### 2.1. Strains, Culturing Conditions

The *A. nidulans* THS30.3 reference strain and the *ΔmnSOD* (*ΔsodB*) mutant, with *sodB* gene deletion (AN5577; [5]), were maintained on Barratt’s minimal nitrate agar plates [11] at 37 °C, and freshly grown conidia from 6-day cultures [5] were used in further experiments.

For submerged cultivation, 500 mL Erlenmeyer flasks containing 100 mL of Barratt’s minimal nitrate broth were inoculated with 5 × 10^7^ conidia and incubated at 37 °C and at 3.7 Hz shaking frequency for 16 h [5]. Stress exposure with 0.16 mM menadione sodium bisulfite (MSB) was carried out at 16 h of incubation time (exponential growth phase culture; [5]), and mycelial samples were taken at 30 min (for RNA isolation and determination of superoxide formation) and 5 h (for measuring SOD activities) after the stress treatment.

For surface cultivation, Barratt’s minimal nitrate agar plates supplemented with 0 or 0.025 mM MSB were point-inoculated with 5 μL of conidia suspension (2 × 10^7^ conidia/mL; [5]). The cultures were incubated at 37 °C for 5 days, and the diameters of the colonies were measured.

### 2.2. Measuring Superoxide Formation and Superoxide Dismutase (SOD) Activities

The intracellular superoxide levels were determined by the formation of ethidium (Et) from dihydroethidium as described earlier [12]. The formed Et was quantified spectrofluorimetrically and was expressed on the dry cell mass (DCM) of the samples. Total SOD activities were measured spectrophotometrically by the rate assay of Oberley and Spitz [13] with cell-free extracts prepared by X-pressing [12]. Protein content of the samples was determined with Bradford’s reagent.

### 2.3. Reverse-Transcriptional Quantitative Real-Time Polymerase Chain Reaction (RT-qPCR) Assays

Total RNA was isolated from lyophilized mycelia according to Chomczynski [14]. RT-qPCR assays were performed with an Xceed qPCR SG 1-step 2x Mix Lo-ROX Kit (Applied Biotechnologies, Praha, Czech Republic) following the manufacturer’s instructions using the primer pairs listed in Table S1. The AN9168 gene (encoding a putative glycerol transporter) was used as reference (Table S1) to characterize relative transcription levels using ΔCP (difference between the crossing point of the reference and target genes within a sample) values.

### 2.4. High-Throughput RNA Sequencing

Lyophilized mycelia were used to isolate total RNA by the Trisol method [14]. Samples were taken from the following four cultures with three biological replicates:

(1) Untreated cultures of *A. nidulans* THS30.3 reference strain;
(2) MSB (0.16 mM) stress-treated cultures of *A. nidulans* THS30.3 reference strain;
(3) Untreated cultures of *A. nidulans ΔsodB* mutant;
(4) MSB (0.16 mM) stress-treated cultures of *A. nidulans ΔsodB* mutant

MSB was added to exponential-growing-phase cultures at 16 h, and samples were taken after 30 min of incubation.

RNA sequencing (from library preparation to generation of fastq.gz files) was carried out at the Genomic Medicine and Bioinformatic Core Facility, Department of Biochemistry and Molecular Biology, Faculty of Medicine, University of Debrecen, Debrecen, Hungary. RNA libraries for single-read 75 bp Illumina RNA sequencing were prepared using a TruSeq RNA Sample preparation kit (Illumina, San Diego, CA, USA) according to the manufacturer’s protocols. All 12 library pools were sequenced in the same lane of a sequencing flow cell. The obtained reads varied between 22.2 and 36.5 million reads per sample. Reads were aligned to the genome of *A nidulans* FGSC A4 (https://fungidb.org/common/downloads/Current_Release/AnidulansFGSCA4/fasta/data/FungiDB-56_AnidulansFGSCA4_Genome.fasta and https://fungidb.org/common/downloads/Current_Release/AnidulansFGSCA4/gff/data/FungiDB-56_AnidulansFGSCA4.gff; both accessed on 24 October 2023) using hisat2 software (version 2.1.0) [15]. In the case of each sample, >94% of reads were successfully aligned. Count values were generated with featureCounts software (Version 2.0.0) [16]. Differential expression analysis of the read counts was performed using DESeq2 (version 1.34.0) [17]. RPKM (reads per kilobase per million mapped reads) values were calculated with the “rpkm” function of the edgeR package [18], and PCA (principle component analysis) was performed with the “prcomp” function.

### 2.5. Evaluation of Transcriptome Data

When comparing two groups, upregulated and downregulated genes were defined as differentially expressed genes (adjusted *p*-value < 0.05) where log_2_FC > 1 or log_2_FC < −1, respectively. FC stands for “fold change”, and log_2_FC stands for the log_2_FoldChange numbers calculated by DESeq2 software (version 1.34.0) using the untreated cultures or the THS30.3 cultures as reference.

Gene set enrichment analyses, performed on the ShinyGO 0.77 (http://bioinformatics.sdstate.edu/go/; accessed on 24 October 2023) platform applying default settings, were used to characterize the composition of selected gene sets. Terms containing fewer than three genes or hits with only one gene were omitted from the analysis, and only hits with a corrected *p*-value < 0.05 were taken into consideration during evaluation. Since gene set enrichment analyses highly depend on the size of the studied gene sets, the analyses for differentially expressed genes (DEGs) were performed with different log_2_FC thresholds: log_2_FC > 0, log_2_FC > 1 (“upregulated genes”), log_2_FC > 2, and log_2_FC < 0, log_2_FC < −1 (“downregulated genes”), log2FC < −2.

The enrichment of genes belonging to the “Fe-S cluster assembly”, “Antioxidant enzyme”, “Respiration”, and “Sterigmatocystin cluster” gene groups was tested by Fisher’s exact test (“fisher.test” function of R project; www.R-project.org/; accessed on 24 October 2023). “Fe-S cluster assembly” genes were defined as genes involved in [2Fe-2S] cluster assembly, [4Fe-4S] cluster assembly, iron–sulfur cluster assembly, iron–sulfur cluster assembly, protein maturation by [2Fe-2S] cluster transfer, protein maturation by [4Fe-4S] cluster transfer, and protein maturation by iron–sulfur cluster transfer according to the data available at FungiDB (https://fungidb.org/fungidb/app; accessed on 24 October 2023). Antioxidant enzyme genes were genes encoding or putatively encoding enzymes with observed/predicted catalase, cytochrome c peroxidase, glutathione disulfide oxidoreductase, glutathione peroxidase, glutathione transferase, peroxidase, peroxiredoxin, superoxide dismutase and thioredoxin-disulfide reductase activities according to the data available at FungiDB (https://fungidb.org/fungidb/app; accessed on 24 October 2023). Sterigmatocystin cluster genes were collected from the paper of Inglis et al. [19]. The “Respiration” gene group contains known and putative NADH dehydrogenase, ubiquinol-cytochrome c reductase, cytochrome c, cytochrome c oxidase and alternative oxidase genes according to the FungiDB (https://fungidb.org/fungidb/app; accessed on 24 October 2023). In the case of stress genes, the *A. nidulans* stress gene set described by the Fungal Stress Response Database (FSRD) [20] was used.

## 3. Results

### 3.1. Intracellular Superoxide Formation and SOD Activity

**Figure 1:**
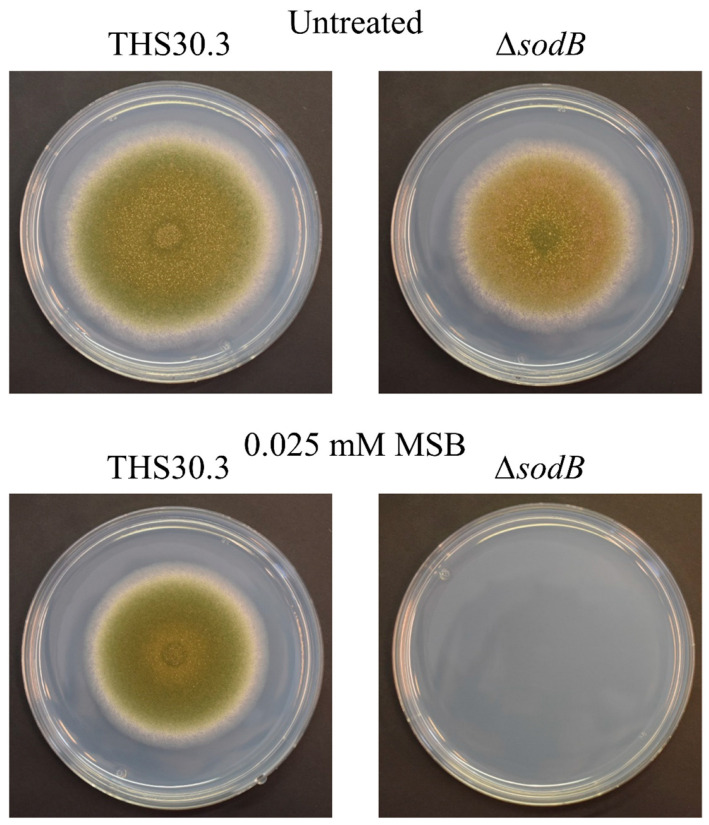
MSB stress tolerance of a *∆sodB* gene-deletion mutant and the appropriate reference (THS30.3) *A. nidulans* strain on agar plates.

**Figure 2:**
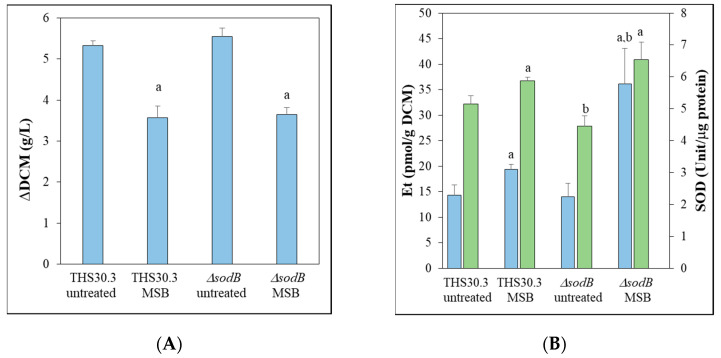
Changes in the DCM (**A**) as well as Et production and specific SOD activities (**B**) after MSB treatment in submerged *A. nidulans* cultures. *A. nidulans* cultures (THS30.3 wild type and *∆sodB* gene-deletion mutant) were treated with 0.16 mM of MSB. The means ± SDs calculated from three biological replicates are presented. On (**B**), blue and green columns indicate Et production and total SOD activities, respectively. a—Significant difference between MSB-treated and untreated cultures (Student’s *t*-test; *p* < 0.05). b—Significant difference between the mutant and the reference strain (Student’s *t*-test; *p* < 0.05).

The importance of mitochondrial MnSOD/SodB [5] during MSB-induced oxidative stress was studied in *A. nidulans* using genome-wide transcription data recorded by RNA sequencing. MSB-treated and untreated submerged cultures of a *sodB* gene-deletion mutant (*ΔsodB*) and its reference strain (THS30.3) were included in this study. Both strains were much less sensitive to MSB stress in submerged cultures than on stress agar plates, and it was particularly true for the *ΔsodB* mutant (Figure 1 and Figure 2A). Furthermore, the growth inhibitory effect of MSB was quite similar in the case of the submerged cultures of THS30.3 reference and *ΔsodB* strains (Figure 2A). MSB treatment increased Et production (characteristic for superoxide formation; [21]) significantly in both strains (Figure 2B). The Et production values were significantly higher in the MSB-treated cultures of the *ΔsodB* mutant than in the reference strain (Figure 2B), suggesting that despite the similar growth profiles (Figure 2A), MSB treatment disturbed redox homeostasis more strongly in the mutant. Although the specific SOD activities were significantly lower in the *ΔsodB* mutant in untreated cultures, after MSB treatment, no significant differences were observed (Figure 2B).

### 3.2. Genome-Wide Transcriptional Consequences of MSB Treatment and Deletion of the sodB Gene in A. nidulans

MSB treatment and *sodB* gene deletion substantially altered the transcriptome in both strains, and the consequences of MSB treatment were greater than those of *sodB* gene deletion (Figure S1). The biological replicates had well-matched transcriptomes as indicated by principal component analyses (Figure S1), and the RNAseq data showed good positive correlation with the RT-qPCR data of the selected genes (Table S1). In the case of the reference strain, the transcriptional changes caused by MSB stress showed a strong positive Pearson’s correlation (correlation coefficient = 0.73; Table S2) with our previous dataset recorded with 60-mer oligonucleotide high-density DNA chips (GSE63019; Gene Expression Omnibus; GEO; http://www.ncbi.nlm.nih.gov/geo/, accessed on 24 October 2023 [22,23,24].

**Figure 3:**
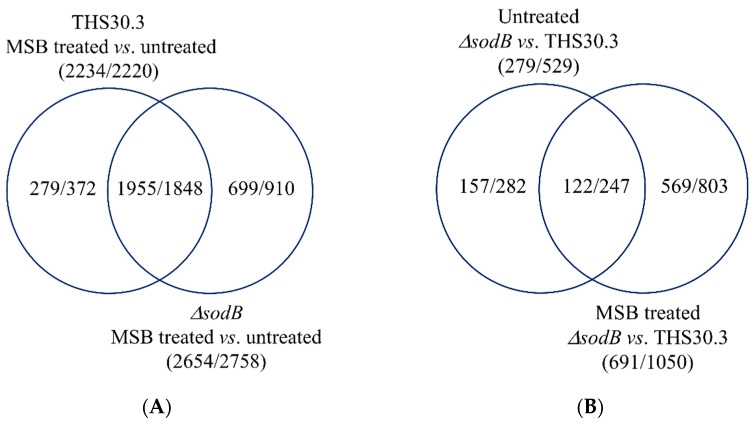
Venn analysis of the genome-wide transcriptional changes elicited by MSB treatment or *sodB* gene deletion. The numbers of upregulated/downregulated genes are presented. Upregulated and downregulated genes were defined as DEGs where log_2_FC > 1, and log_2_FC < −1, respectively. (**A**): Effect of MSB treatment on the wild-type strain (THS30.3) and the *∆sodB* mutant. (**B**): Consequences of *sodB* gene deletion in untreated and MSB (0.16 mM)-treated cultures.

**Figure 4:**
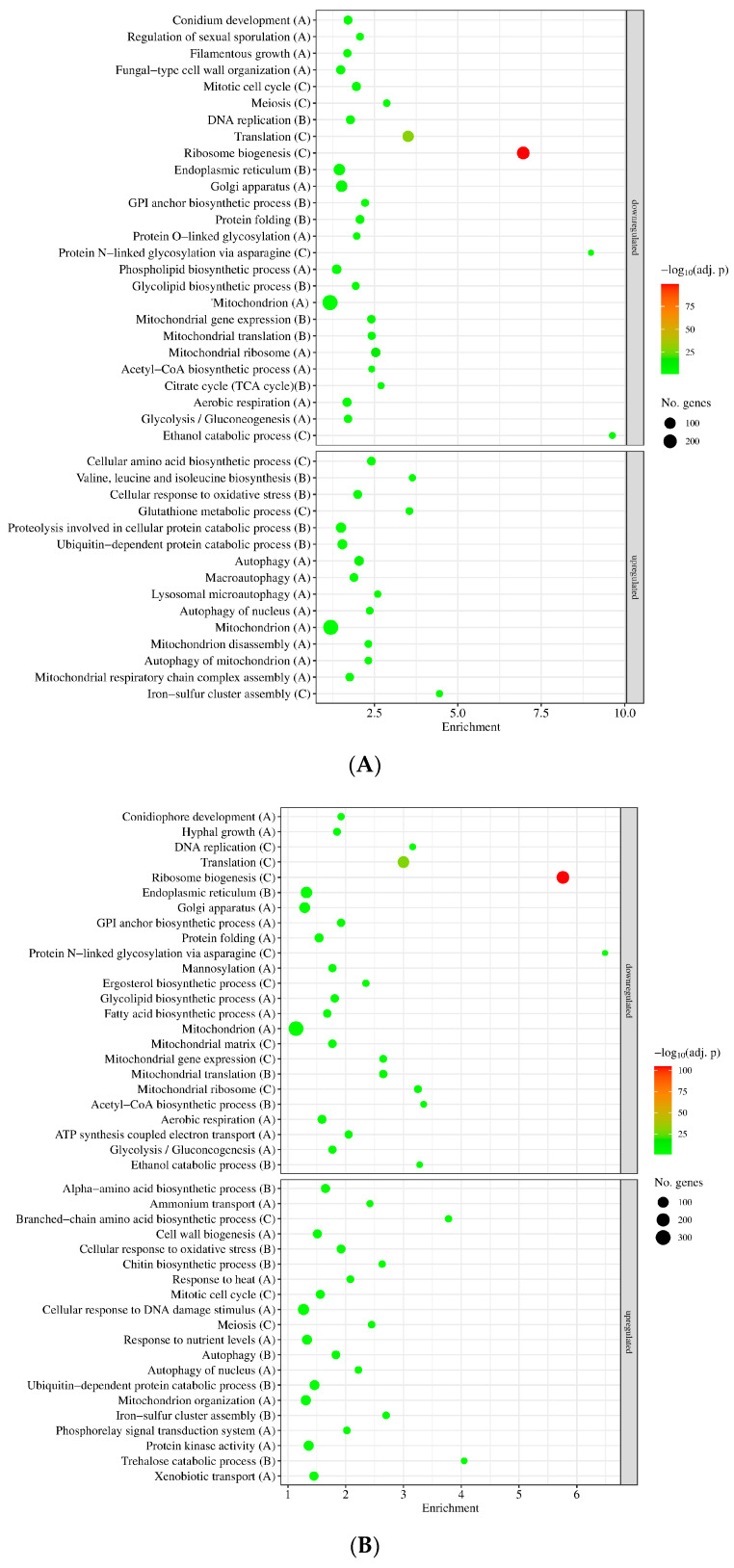
Gene set enrichment analyses of the effect of MSB treatment on the reference strain (THS30.3; (**A**)) and the *∆sodB* mutant (**B**). Selected significantly enriched (*p* adjusted < 0.05) GO terms are presented. The full list of the enriched GO terms are available in Table S3. Letters in parentheses indicate the studied gene set: “A”—all DEGs, “B”—DEGs with |log_2_FC| > 1, “C”—DEGs with |log_2_FC| > 2. If a selected term was enriched in more than one gene set, only the set with the strongest criteria is presented.

The numbers of MSB stress-responsive genes were similar in the two strains (Figure 3A), concurring well with the observation that *sodB* gene deletion did not cause substantial difference in MSB-induced growth inhibitions in submerged cultures (Figure 2A). The overlap between the two MSB stress-responsive gene sets was also substantial: Out of the MSB stress-responsive genes of the mutant, 1955 (74%) upregulated genes and 1848 (67%) downregulated genes showed stress responsiveness in the reference strain as well (Figure 3A). Gene set enrichment analyzes suggested substantial overlap between the two stress responses too (Figure 4, Table S3). Vegetative growth-related processes (e.g., mitotic cell cycle, DNA replication, translation, ribosome biogenesis, cell wall biogenesis), endoplasmic reticulum (ER), Golgi and mitochondrion functions, as well as glucose utilization were downregulated, while autophagy, ubiquitin-dependent protein catabolic process, oxidative stress response genes and mitochondrion assembly genes were upregulated in both strains (Figure 4, Table S3). Fe–S cluster assembly genes, antioxidative enzymes genes (e.g., *catA* and *catB* catalase and *glrA* glutathione reductase genes) and other stress genes were also upregulated by MSB treatment in both strains (Tables S1 and S4). Both *sodA* (AN0241) and *sodB* (AN5577) genes were upregulated under MSB stress in the reference strain (Table S4). Deletion of the *sodB* gene did not result in upregulation of any known or putative antioxidative enzyme genes (including the *sodA*, *sodM* and AN1131 SOD genes) tested (Tables S1 and S4). However, certain genes like *catB* catalase and AN5440 putative cytochrome c peroxidase genes showed strong upregulation in the mutant after MSB treatment, and consequently the transcriptional activity of these genes was at least twice as high in the *∆sodB* mutant than in the reference strain in the presence of MSB (Tables S1 and S4). Downregulation of mitotic cell cycle, ribosome biogenesis, translation, citrate cycle and endoplasmic reticulum to Golgi vesicle-mediated transport genes as well as the upregulation of iron–sulfur cluster assembly and antioxidative enzyme genes were also observed previously with the reference strain using a DNA chip approach [23].

**Figure 5:**
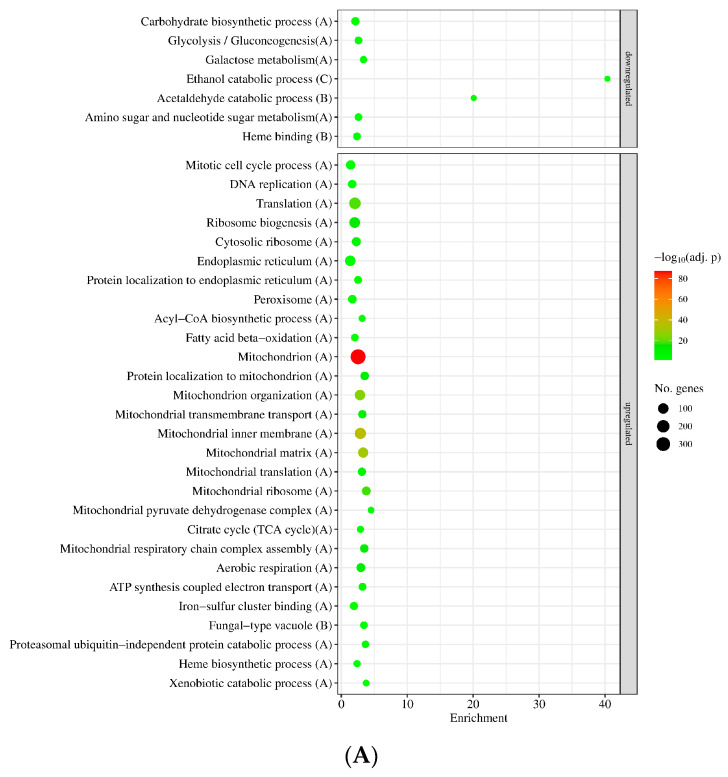

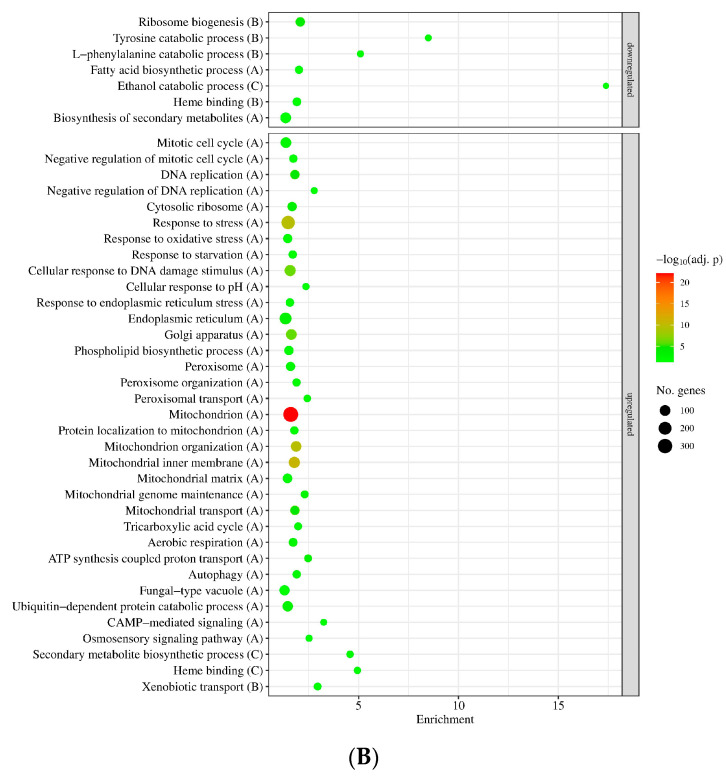
Gene set enrichment analyses of the consequences of *sodB* gene deletion in untreated (**A**) and MSB-treated (**B**) cultures. Selected significantly enriched (*p* adjusted < 0.05) GO terms are presented. The full list of the enriched GO terms is available in Table S3. Letters in parentheses indicate the studied gene set: “A”—all DEGs, “B”—DEGs with |log_2_FC| > 1, “C”—DEGs with |log_2_FC| > 2. If a selected term was enriched in more than one gene set, only the set with the strongest criteria is presented.

Despite the similarities between the two stress responses, the difference between the transcriptomes of the reference strain and the *ΔsodB* mutant increased by the stress treatment from 279 + 529 = 808 genes (untreated cultures) to 691 + 1050 = 1741 genes (MSB-treated cultures) (Figure 3B) suggesting some differences between the stress responses of the strains. Gene set enrichment analyses showed that more or less similar processes showed differences between the mutant and the reference strain under the tested two (MSB-exposed and unstressed) culture conditions. Mitotic cell cycle and DNA replication genes, ER and Golgi-dependent processes, protein catabolism as well as mitochondrion organization and mitochondrial functions were upregulated in the mutant relative to the reference strain both in untreated and MSB-treated cultures (Figure 5, Table S3). In contrast, genes of the “Negative regulation of mitotic cell cycle” and “Negative regulation of DNA replication” GO terms as well as “peroxisome” and “stress response” genes were enriched in the upregulated gene set only in the presence of MSB, while “Ribosome biogenesis” GO term genes were enriched in the upregulated gene set in the absence of MSB but enriched in the downregulated gene set under MSB exposure in the *∆sodB* vs. THS30.3 comparison (Figure 5, Tables S3 and S4).

It is noteworthy that genes related to the “Aerobic respiration” GO term were enriched in the downregulated gene sets by MSB treatment in both strains, meanwhile they were enriched in the upregulated gene sets after deletion of *sodB* (*∆sodB* mutant vs. THS30.3 reference strain) both in the presence and absence of MSB (Table S3). In contrast, several genes encoding/putatively encoding NADH dehydrogenase or cytochrome c oxidase subunits were upregulated by MSB treatment, and many of them also showed upregulation in the *sodB* gene-deletion mutant when compared to the reference strain irrespectively of the presence of MSB (Table S4). Furthermore, the *aodA*/*aoxA* (AN2099, alternative oxidase) gene showed MSB-dependent upregulation in both strains, and no significant difference between the *aodA*/*aoxA* transcriptional activities of the two strains was detected independently of MSB (Table S4).

Importantly, genes of the “Conidium development” GO term, including *stuA* (AN5836) encoding a transcription factor regulating conidiophore morphogenesis [25], were enriched in the downregulated gene set of both strains when MSB-treated and untreated cultures were compared (Figure 4, Table S3). The *stcK* gene (AN7814) encoding a polyketide synthase of the sterigmatociystin gene cluster was downregulated in the *∆sodB* mutant relative to the reference strain both in the presence and absence of MSB (Table S4). Sterigmatocystin cluster genes were enriched in the downregulated gene set when the transcriptomes of the *∆sodB* mutant and the reference strain were compared in the presence of MSB, but no significant enrichment was observed in any other comparisons tested (Table S4).

## 4. Discussion

Fungal MnSODs play a complex role in oxidative stress defense via maintaining the integrity and functions of mitochondria under both endogenous and exogenous superoxide stresses [2,3,4,5]. Not surprisingly, MnSODs have been reported as key elements in the regulation of versatile physiological processes responding to changing intracellular reactive oxygen species levels, including aging and stationary growth phase survival [2,26,27,28], invasion of hosts by pathogenic fungi [2,4,29,30,31,32] and the production of harmful mycotoxins [5,10,33,34]. Importantly, fungal MnSODs also play important roles in asexual sporulation and the preservation of conidia under various environmental stress conditions, including heat, cold (during storage) and oxidative stresses [5,9,31].

The *A. nidulans sodB* gene encodes a mitochondrial MnSOD (SodB) enzyme, which is important in the protection of mitochondria against the deleterious effects of both endogenous and exogenous oxidative stress [5]. As expected, deletion of the *sodB* gene was accompanied by increased sensitivity to the superoxide-generating agent MSB on agar plates ([5]; Figure 1). It is worth noting that the MSB stress tolerance of the fungus was much higher in submerged cultures (Figure 2), and it was particularly true for the *ΔsodB* mutant, which was unable to grow at 0.025 mM MSB on the surface stress agar culture but showed only approximately a 35% growth reduction in submerged cultures at a 0.16 mM MSB concentration (Figure 2A).

The major difference between the two types of experiments (stress agar vs. submerged liquid) is that conidia have to germinate in the presence of MSB in stress-exposed surface cultures, while only growing pelleted mycelia sense MSB stress in submerged cultures. Previous studies demonstrated that (i) fungal MnSODs are necessary in the maintenance of conidia under environmental stress conditions [5,9,31], and (ii) mitochondrial activity even increases markedly during germination [35,36]. Because Fe–S cluster proteins of mitochondria are sensitive to superoxide stress [37], the elevated mitochondrial activity during germination can be accompanied by increased O_2_^●−^ formation and, as a consequence, by an increased need for superoxide-neutralizing enzymes like SodB. Therefore, the decreased stability of conidia under oxidative stress in general and the increased mitochondrial activity during germination together may explain the increased MSB stress sensitivity observed on surface cultures, and these processes may also elucidate why the *ΔsodB* mutant was much more sensitive to MSB stress in surface agar cultures than the reference strain. In good accordance with this hypothesis, increased *tert*-butylhydroperoxide (generates lipid peroxides) and heavy metal (Cu^2+^, Cd^2+^) tolerances were recorded when *A. nidulans* mycelial mats were pre-grown on cellophane sheets on unstressed agar plates (“cellophane colony harvest cultures”), lifted and transferred onto stress agar plates [38,39].

Not surprisingly, *sodB* was upregulated by MSB treatment in liquid submerged cultures (Tables S1 and S4), and deletion of *sodB* caused detectable loss of specific SOD activity of mycelia which was accompanied by increased superoxide production after MSB treatment (Figure 2B). These data demonstrate that SodB was also involved in the protection of mitochondria when exponentially growing mycelia had to cope with MSB stress. In line with this, *A. fumigatus AfSOD2* was highly expressed in growing mycelium at later, 20–30 h incubation times in addition to conidia [9].

Somewhat contradicting the above, the MSB stress-induced growth reduction of the *ΔsodB* mutant was similar to that of the reference strain in submerged cultures (Figure 1A) and the MSB stress-elicited transcriptional changes of the two strains were also similar both in their intensity and nature (Figure 3 and Figure 4, Table S3). One explanation of this unexpected behavior is that the relatively small differences between the transcriptional changes of the two strains were enough for the *ΔsodB* mutant to overcome the disadvantages caused by gene deletion. Indeed, certain antioxidative enzyme genes, like *sodA* (cytosolic CuZnSOD), *ccp1* and AN5440 (verified and putative cytochrome c peroxidase genes), *catB* (catalase) and *trxR* (thioredoxin reductase) as well as *tpxB* and *trxA* (thioredoxin-dependent peroxidase genes) showed significantly higher transcriptional activity in the *ΔsodB* mutant than in the reference strain after MSB treatment (Tables S1 and S4), and no significant difference was observed between the specific SOD activities of the strains (Figure 2B). Moreover, several stress genes, like oxidative stress, starvation stress, pH stress, ER stress, DNA damage stress genes enriched in the upregulated gene set of only the *ΔsodB* mutant after MSB treatment (Table S3, Figure 5).

It is reasonable to assume that exogenous or endogenous antioxidants and/or up-regulation of mitochondrial quality control genes can mitigate the disadvantageous physiological consequences of the ablation of MnSOD genes [8,40,41]. For example, primaquine (an antimalarial drug) sensitivity of the *S. cerevisiae Δsod2* mutant could be counterbalanced by the addition of ascorbate and *N*-acetyl cysteine as well as by the overexpression of *AIM32* (coding for a 2Fe–2S mitochondrial protein contributing to redox quality control) and *MCR1* (encoding mitochondrial NADH-cytochrome b5 reductase) genes [40]. Furthermore, any blockage of the glutathione/glutaredoxin and thioredoxin redox systems [42,43] may intensify the effects of environmental stress on the MnSOD gene-deletion mutants. In accordance with this, targeted repression of baker’s yeast *SOD2* increases ethanol sensitivity (not in respiration-deficient strains) via preventing the ethanol-induced relocalization of Yap1 resulting in decreased Trx2 (cytoplasmic thioredoxin isoenzyme) and Gsh1 (γ-glutamylcysteine synthetase, catalyzing the first reaction of glutathione biosynthesis) protein levels [41].

Upregulation of DNA damage stress genes in the *A. nidulans ΔsodB* mutant was also foreseeable because Baker’s yeast Sod2 together with Sod1 (encodes cytosolic CuZnSOD) and Ccs1 (the copper chaperone for superoxide dismutase Sod1) profoundly contributes to the preservation of the integrity of the nuclear DNA in replicatively old yeast cells [44]. Furthermore, in the fission yeast *Schizosaccharomyces pombe*, deletion of *sod2* decreases chronological life span via increasing nuclear DNA mutation frequency and accelerating the degradation of cellular proteins and nuclear DNA with concurrent cell death [45].

Another alternative explanation for the relatively small differences between the MSB stress responses of the *ΔsodB* mutant and the THS30.3 control strains can be that prevention of mitochondrial damages is not strictly essential under these experimental conditions. Decreasing mitochondrial activity, replacing damaged mitochondrial proteins and elimination of unrepairable mitochondria can also be important and adequate responses to successfully adapt to stresses threatening mitochondrial integrity and functions [6,8]. Transcriptional changes demonstrate that MSB stress reduced the activity of mitochondrial functions (e.g., mitochondrial translation, aerobic respiration and acetyl-CoA biosynthesis genes were downregulated; Figure 4, Table S3), upregulated mitochondrial assembly processes (e.g., mitochondrial respiratory chain complex assembly genes in the reference strain and mitochondrion organization genes in the gene-deletion mutant, while iron–sulfur cluster assembly genes in both strains showed upregulation; Figure 4, Table S3) as well as mitochondrion disassembly and/or autophagy genes (Figure 4, Table S3).

A previous study by Leiter et al. [5] demonstrated that the deletion of *A. midulans sodB* gene negatively affects the total, KCN-sensitive cytochrome c-dependent (the main source of endogenous O_2_^●−^) and AOX (alternative oxidase)-dependent alternative respirations only in carbon-starving cultures of the fungus, which was clearly indicative of significantly decreased mitochondrial activity under these experimental conditions. In glucose-supplemented cultures, *sodB* deletion influences the KCN + SHAM-resistant, residual (not cytochrome c and not alternative oxidase-dependent; [5,46,47] respiration only [5]. In our experiments, *sodB* deletion even resulted in higher-level expression of the aerobic respiration genes (but not the *aodA/aoxA* alternative oxidase gene) in relation to the THS30.3 reference strain independently of MSB stress in glucose-supplemented cultures (Tables S3 and S4). These upregulations may have helped the *∆sodB* mutant to maintain respiration activity at the level of the reference strain in the presence of glucose. These findings challenge the view that *sodB* deletion alone would threaten mitochondrial integrity and function (including respiration) in glucose-supplemented, growing, liquid-submerged cultures.

Similarly, *sodB* deletion increases sterigmatocystin production only in carbon-starving cultures [5], and this was also reflected in this study by the insensitivity of the biosynthetic gene cluster to *sodB* deletion and MSB exposure. Although *SodB* is necessary for conidiogenesis and the maintenance of conidia in surface cultures [5], the general downregulation of conidium development genes is foreseeable in liquid submerged cultures where the formation of asexual spores is blocked [48].

In summary, we can speculate that although upregulation of *sodB* expression in the reference strain was an important element of the oxidative stress response, it was far from sufficient to prevent mitochondrion damages under MSB stress. Because the significance of other mitochondrial quality control and maintenance processes (e.g., replacement of damaged proteins, decreasing mitochondrial activity and elimination of unrepairable mitochondria by mitophagy) seems to exceed that of SodB in the prevention of the deleterious consequences of MSB exposure, the overall physiological consequences of *sodB* deletion were smaller than expected. This was also nicely reflected by the similar transcriptomic changes recorded in MSB-exposed submerged liquid cultures of the *ΔsodB* mutant and the reference strains. On the other hand, the activity of mitochondria was intensified under germination of conidia when the downregulation of mitochondrial processes and the replacement of damaged mitochondrial proteins can be a far less efficient strategy than preventing mitochondrial damages by antioxidative enzymes, including SodB. Consequently, *sodB* deletion triggered more spectacular MSB sensitivity phenotypes in stress agar surface cultures [5]. To verify the significant role of SodB in the maintenance of conidia and vegetative tissue [5,9] in *A. nidulans* stress agar cultures, mycelial and conidial transcriptomes will be mapped and compared in both unstressed and MSB-treated cultures of the *ΔsodB* mutant and THS30.3 control strains [49].

Our results demonstrate the superior flexibility of the Aspergilli to adapt to versatile environmental stress conditions and support the view that germination is a critical part of the fungal life cycle. Therefore, future antifungal strategies should focus more on killing germinating conidia rather than eliminating growing hyphae when possible [50,51].

Because the role of SodB in the stabilization of mitochondrial functions and facilitating hyphal growth under superoxide stress seems to be less than expected in glucose-supplemented liquid submerged cultures, further research should focus on the hypothesized role of this superoxide dismutase in the stationary phase survival of *A. nidulans* similar to yeasts and other filamentous fungi [2,26,27,28]. We suggest that the effects of *sodB* deletion and overexpression on the vitality and secondary metabolite yields of carbon-starved and carbon-limited *A. nidulans* liquid submerged cultures [5] should be studied in-depth in the future using different omics techniques.

## Data Availability

The transcriptome data sets are available in the Gene Expression Omnibus database (GEO; http://www.ncbi.nlm.nih.gov/geo/; accessed on 24 October 2023) with the following accession number: GSE244381.

## Supplementary Materials

The following supporting information can be downloaded at: https://www.mdpi.com/article/10.3390/jof9111060/s1, Figure S1: Principal component (PC) analysis of the RNAseq data obtained from MSB-treated and untreated cultures of *A. nidulans* THS30.3 (reference strain) and *∆sodB* mutant, Table S1: RT-qPCR data of selected genes, Table S2: Correlation between RNAseq and DNA chip data, Table S3: Results of the gene set enrichment analyses, Table S4: RNAseq data of “Fe-S cluster assembly”, “Antioxidant enzyme”, “Respiration”, and “Sterigmatocystin cluster” genes.
