# Supplementary material for: Global Transcriptomic Changes Elicited by sodB Deletion and Menadione Exposure in Aspergillus nidulans
Source: J Fungi (Basel). 2023 Oct 30;9(11):1060. doi: 10.3390/jof9111060 (PMC10671992; doi:10.3390/jof9111060)
Supplement: Supplementary file 1 [file jof-09-01060-s001.zip › Fig S1.pdf]

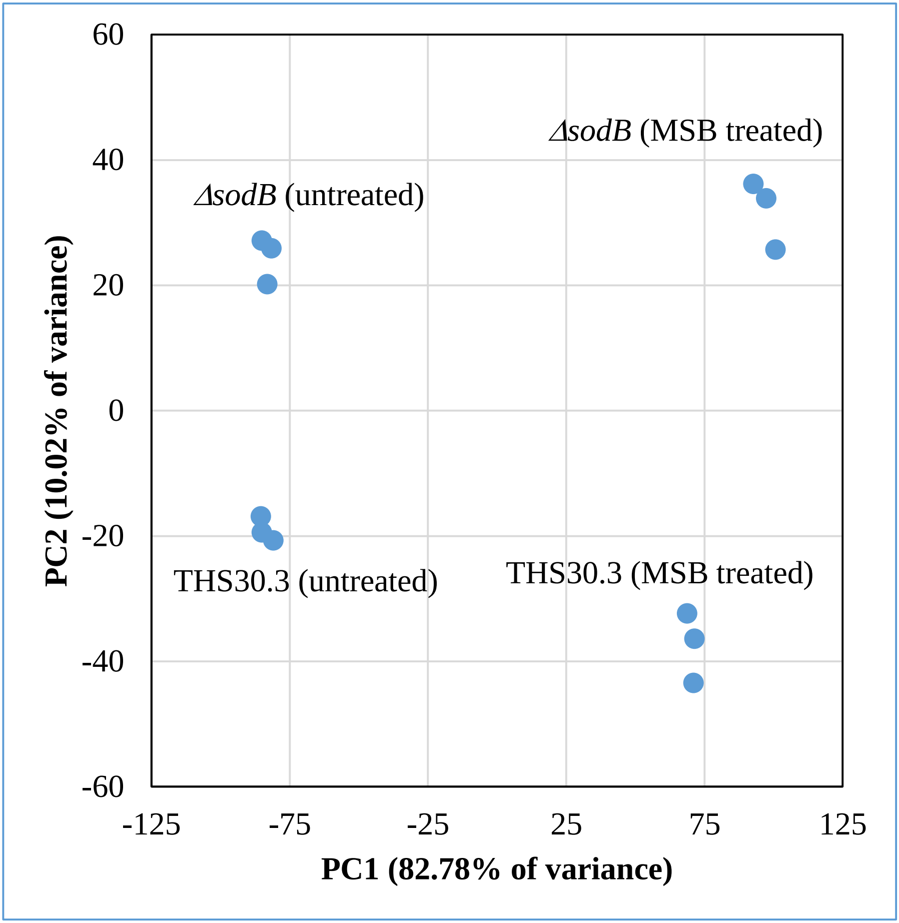

Figure S1 Principal component (PC) analysis of the RNAseq data obtained from MSB treated and untreated cultures of the *A. nidulans* THS30.3 (reference strain) and *ΔsodB* mutant.

The analysis was carried out using the rlog data generated by the DESeq2 software.
